# A Rare Manifestation of Mycobacterium kansasii as a Cutaneous Infection: A Case Report

**DOI:** 10.7759/cureus.111975

**Published:** 2026-07-03

**Authors:** Wandera M Carey, Antony Kimani, Zamanali Khakhar, Dheer A Kalaiya, Alok Iyer, Sayed K Ali

**Affiliations:** 1 Department of Internal Medicine, Aga Khan University Hospital, Nairobi, KEN; 2 Department of Medicine, University of Nairobi, Nairobi, KEN

**Keywords:** acquired immune deficiency syndrome (aids), cutaneous nontuberculous mycobacterial infection, extrapulmonary mycobacterial infection, mycobacterium kansasii, nontuberculous mycobacteria (ntm)

## Abstract

Nontuberculous mycobacteria (NTM) are environmental organisms increasingly recognized as opportunistic pathogens, particularly in immunocompromised individuals. *Mycobacterium kansasii*, a slow-growing photochromogen (Runyon group I), is primarily associated with pulmonary disease; however, extrapulmonary and cutaneous manifestations are uncommon and often underdiagnosed. Given the rising incidence of NTM infections, cutaneous disease should be considered in the differential diagnosis of skin lesions, especially in immunocompromised patients. Early microbiological confirmation is crucial for appropriate therapy, as *M. kansasii* is intrinsically resistant to pyrazinamide. Herein, we present a case of pathologically confirmed cutaneous *M. kansasii* infection occurring in isolation, illustrating the diagnostic challenges and therapeutic outcomes.

## Introduction

Cutaneous mycobacterial infection is an uncommon presentation of extrapulmonary tuberculosis (TB) and accounts for less than 2% of all extrapulmonary manifestations [1]. While most cutaneous mycobacterial infections are attributed to the *Mycobacterium tuberculosis *complex, nontuberculous mycobacteria (NTM) are increasingly recognized as an important and underdiagnosed cause of skin disease. NTM are mycobacterial species other than those belonging to the *M. tuberculosis *complex. They are generally associated with four major clinical syndromes: pulmonary disease, superficial lymphadenitis, disseminated disease in severely immunocompromised patients, and infections of the skin, soft tissues, bones, and joints [2].

Their nonspecific and highly variable clinical manifestations mimic diverse skin conditions and pose a significant diagnostic challenge to clinicians. Identification of the causative organism is frequently difficult using routine diagnostic tools, contributing to delayed or incorrect diagnoses. According to a study in China, up to half of the patients were misdiagnosed because of the infrequent and difficult identification of the bacillus using common testing tools, as well as the diverse morphology of the lesions, which could present as patches, plaques, or papules. These lesions are often mistaken for psoriasis, discoid lupus erythematosus, erythema nodosum, or pityriasis, among other differential diagnoses [3].

This case aims to contribute to the existing body of literature on cutaneous mycobacterial infections, particularly within the African setting, where published data remain limited.

## Case presentation

A 41-year-old female of African descent, newly diagnosed with HIV infection, with an absolute CD4 count of 7 cells/mm³, presented with a one-month history of painful erythematous nodular lesions involving the right forearm and left leg, with associated joint pain. She had initially presented to an outpatient clinic three weeks prior to admission, where a presumptive diagnosis of erythema nodosum was made, and she was started on corticosteroid anti-inflammatory therapy. Despite treatment, the lesions persisted without improvement. She denied any preceding trauma or injections at the sites of the lesions.

The onset of the skin lesions was preceded by a one-month history of a productive cough with clear sputum. One week prior to admission, she reported experiencing fever associated with generalized body weakness and anorexia. She denied hemoptysis or shortness of breath. She also denied drenching night sweats or unintentional weight loss. She had a notable history of travel across Africa in the preceding year. There was a history of erythema nodosum and shingles, both of which had resolved following treatment at the time of the initial diagnosis. She denied a history of alcohol or cigarette use. She had no known history of contact with individuals with a chronic cough or receiving treatment for TB.

**Figure 1:**
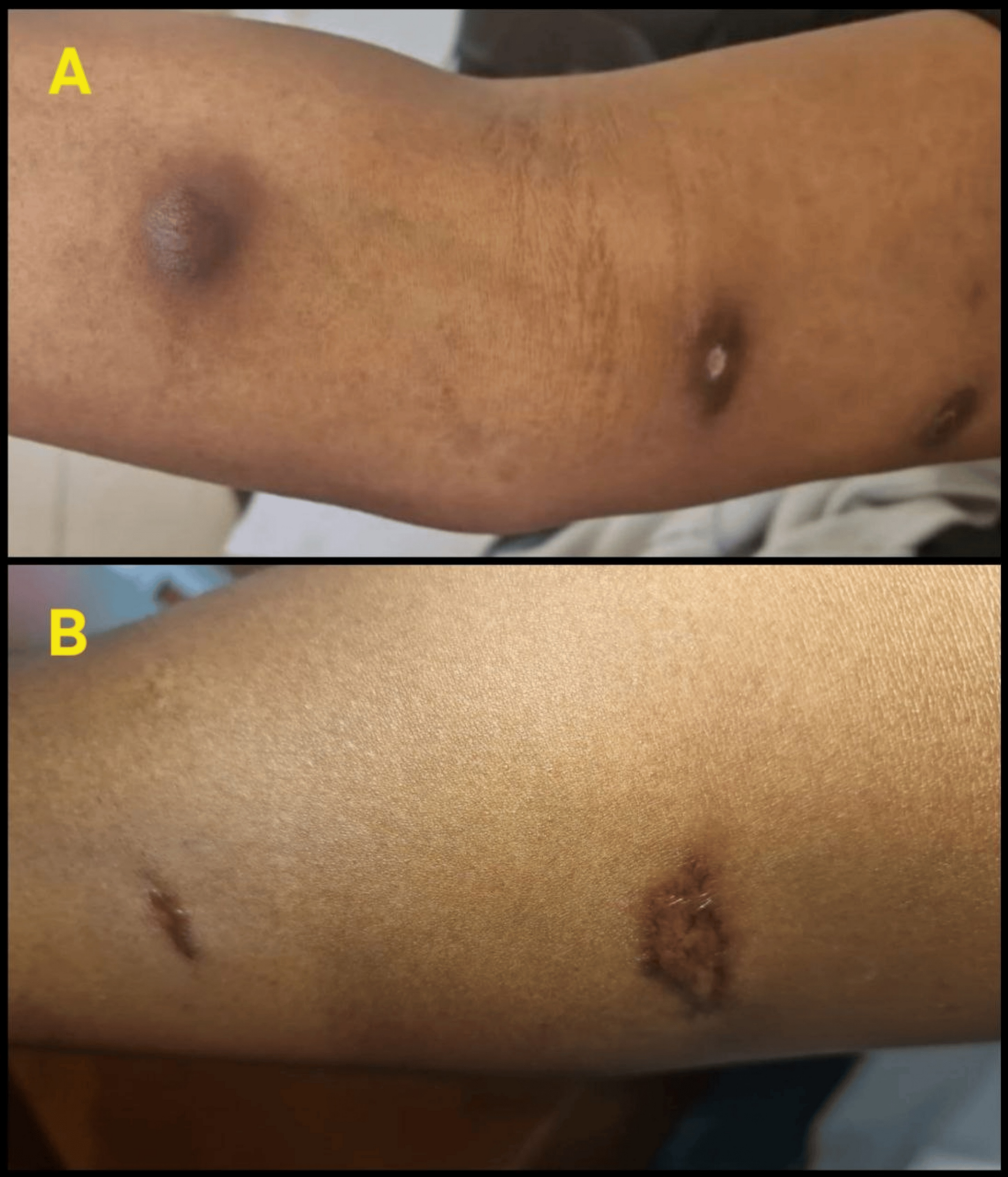
(A) Nodular lesion involving the medial aspect of the arm and proximal forearm with hyperpigmentation of the overlying skin. (B) Healed cutaneous lesions demonstrating residual scar formation and hyperpigmentation at the site of prior involvement.

On examination, she appeared ill, was tachycardic at 132 bpm, and was afebrile. There was no palpable lymphadenopathy, pallor, or jaundice. Oral examination revealed oral thrush involving the hard palate. Skin examination revealed multiple well-circumscribed, hyperpigmented nodular lesions that were nontender with no discharge; however, one lesion on her right arm showed central ulceration (Figure 1). Respiratory examination revealed crepitations at the left lung base. The remainder of the systemic examination was unremarkable.

**Figure 2:**
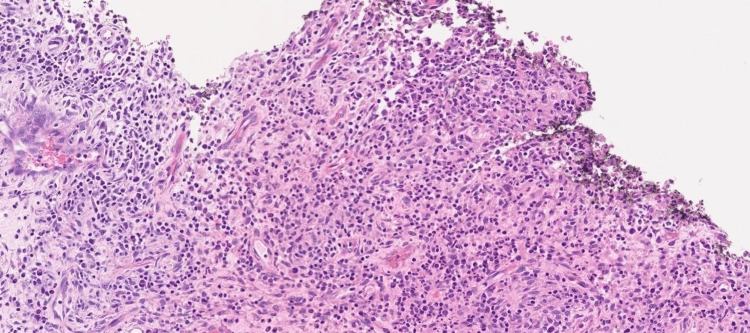
Histopathological findings from a skin biopsy of a nodular lesion on the right arm demonstrating granulomatous inflammation with well-formed epithelioid cell granulomas, multinucleated Langhans giant cells, and central caseating necrosis, consistent with mycobacterial infection and correlated with microbiological findings confirming Mycobacterium kansasii (hematoxylin and eosin stain).

A skin biopsy of the nodular lesion on the right arm was obtained for histopathological examination and mycobacterial studies (Figure 2). Tissue culture yielded a pure isolate of a Mycobacterium species, which was differentiated using the Genotype Mycobacterium Common Mycobacteria (CM) assay and identified as *Mycobacterium kansasii*. Although an AFB antibiogram for slow-growing NTM was recommended, it was not pursued because species identification was considered sufficient to establish the diagnosis and guide management. Bronchoalveolar lavage was negative for acid-fast bacilli (AFB), and no pathogens were isolated on culture. Mycobacterial culture of a lymph node was also negative after six weeks of incubation. 

The results of laboratory investigations are summarized in Table 1. Microbiologic findings are detailed in Table 2.

**Table 1:** Patient laboratory investigations ALP, alkaline phosphatase; ALT, alanine transaminase; AST, aspartate transaminase; GGT, gamma-glutamyl transferase; LDH, lactate dehydrogenase

Test	Result	Reference range
WBC	2.95 × 10⁹/L	4.0-10.0 × 10⁹/L
Neutrophils	77%	40-80%
Lymphocytes	12.50%	20-40%
Hemoglobin	8.5 g/dL	12-15 g/dL
Hematocrit	27%	36-46%
RBC	3.35 × 10¹²/L	3.8-4.8 × 10¹²/L
Platelets	334 × 10⁹/L	150-410 × 10⁹/L
AST	60.9 U/L	0-34 U/L
ALT	32.7 U/L	0-49 U/L
GGT	75 U/L	0-38 U/L
ALP	60.5 U/L	46-116 U/L
Total bilirubin	8.4 µmol/L	5-21 µmol/L
Direct bilirubin	4.5 µmol/L	0-5 µmol/L
Indirect bilirubin	3.9 µmol/L	-
Albumin	43.9 g/L	32-48 g/L
Total protein	88.9 g/L	57-82 g/L
CRP	17.9 mg/L	0-10 mg/L
LDH	246 U/L	120-246 U/L
Peripheral blood film	Mild microcytic hypochromic anemia	-
Absolute CD4 count	7 cells/mm³	456-1492 cells/mm³
CD4/CD8 ratio	0.04	0.68-3.61
HIV-1 RNA (PCR)	1,010,000 copies/mL	20-10,000,000 copies/mL
PCP quantitative PCR	Positive (log 5.29)	-
Rapid plasma reagin	Nonreactive	-
Hepatitis B surface antigen	Negative	-
Hepatitis C antibody	Negative	-
*Toxoplasma* IgG antibody	Negative	-
Antinuclear antibody, nucleolar pattern	Positive	-
DFS70 Ab	Negative	-
Anti-Ro52 Ab (IB)	Negative	-
Jo-1 antibody	Negative	-
SS-A/Ro antibody	Negative	-
SS-B/La antibody	Negative	-
Sm (Smith) antibody	Negative	-
Anti-nRNP/Sm IgG Ab	Negative	-
Scl-70 scleroderma Ab	Negative	-
PM/Scl complex Ab	Negative	-
Anti-PCNA Ab FEIA Com	Negative	-
Ribosomal P protein Ab	Negative	-
Centromere B antibody	Negative	-
Nucleosome antibodies	Negative	-
Histone Ab, qualitative	Negative	-
Mitochondrial M2 Ab	Negative	-
Iron	4.46 µmol/L	5.83-34.5 µmol/L
Transferrin	1.50 g/L	2.50-3.80 g/L
Transferrin saturation	11.83%	20.0-50.0%
Ferritin	3516.00 ng/mL	13-150 ng/mL

**Table 2:** Patient microbiological investigations AFB, acid-fast bacilli; LAM, lipoarabinomannan; MOTT, mycobacteria other than tuberculosis

Test	Result
Skin biopsy culture	MOTT final AFB smear result: AFB 3+; organism identified as *Mycobacterium kansasii*
Urinary LAM antigen	Positive

The patient was initiated on antiretroviral therapy consisting of tenofovir disoproxil fumarate/lamivudine/dolutegravir for newly diagnosed HIV and discharged on ART with cotrimoxazole prophylaxis. Given the initial concern for TB, empiric anti-TB therapy with isoniazid, pyrazinamide, rifampicin, and ethambutol, together with pyridoxine, was initiated while awaiting definitive microbiologic diagnosis. Once *M. kansasii* was confirmed via skin biopsy, therapy was appropriately modified to rifampicin, isoniazid, and ethambutol with pyridoxine, alongside double-dose dolutegravir to account for rifampicin drug interactions. She has remained clinically stable on follow-up, with no development of new lesions. The previously noted cutaneous lesions have resolved, with residual post-inflammatory hyperpigmented scarring.

## Discussion

Extrapulmonary TB, particularly its cutaneous forms, has received growing clinical attention in recent years owing to better understanding and improved diagnostics, resulting in higher detection rates. While cutaneous involvement is also seen in NTM infections such as the *Mycobacterium avium *complex (MAC), reports of primary cutaneous NTM infections remain uncommon. They are increasingly recognized as causes of skin and soft tissue infections, and a high index of suspicion is required, particularly in patients with underlying immunosuppression who present with atypical or persistent cutaneous lesions [4]. In this case, the skin lesions were determined to represent a localized infection, with no microbiological evidence of disseminated *M. kansasii *infection, and showed a marked response to appropriate therapy.

*M. kansasii*, first described in 1953, is a slowly growing NTM that is commonly associated with pulmonary disease, although disseminated and extrapulmonary manifestations may occur in individuals with severe immunosuppression [5]. Among NTMs, *M. kansasii *is considered one of the most virulent and shares several microbiologic and clinical characteristics with *M. tuberculosis*, often mimicking classical TB in both presentation and disease course. Cutaneous disease is the most common extrapulmonary manifestation of *M. kansasii* after pulmonary involvement [6]. Unlike other NTM, *M. kansasii* has not been found in soil or natural water supplies but has been isolated from tap water in areas where the microorganism is endemic [7]. Areas of greatest endemicity in the US include Louisiana, Illinois, Texas, and Florida. A case series of *M. kansasii *infection has also been reported in Spain [8]. It is the second most frequently isolated NTM after MAC [9].

The clinical presentation associated with *M. kansasii *closely resembles that of TB, although *M. kansasii *is generally considered less virulent than *M. tuberculosis*. Common presenting symptoms include fever, night sweats, weight loss, productive cough, and dyspnea. Disseminated disease occurs more frequently in patients with advanced HIV infection and is typically defined by the isolation of *M. kansasii *from sites other than the respiratory system, hilar lymph nodes, or skin. Cutaneous lesions caused by *M. kansasii *may present with a wide range of manifestations, including nodules (often sporotrichoid-like), pustules, verrucous lesions, nodular lymphangitic lesions, erythematous plaques, seromas, cellulitis, ulcers, and abscesses. When cutaneous infections with *M. kansasii *occur, they are usually due to local inoculation at the site of minor trauma rather than hematogenous dissemination and are therefore generally confined to a single anatomic region, typically an extremity [5]. A series of 46 patients with HIV and *M. kansasii *infection in Florida reported a median CD4 count of 34 cells/mm³. Of these patients, 91% had pulmonary infection, and only 8% had extrapulmonary disease without coexisting pulmonary disease [10]. In comparison, our patient had profound immunosuppression with an absolute CD4 count of 7 cells/mm³, likely predisposing her to atypical disease manifestations.

Cutaneous lesions caused by *M. kansasii* must be distinguished from other mycobacterial infections, including cutaneous TB and other NTM infections. Other diagnoses that should be excluded include mycotic infections, treponemal infections, and staphylococcal and streptococcal infections. Diagnosis and identification of the causative organism using conventional methods remain challenging, primarily because of the slow growth of certain strains, the low bacillary burden within lesions, and the requirement for specialized culture conditions [5]. As is the case with many patients, topical debridement and administration of antibiotics for presumed cellulitis may further complicate detection by reducing organism yield. Diagnosis of cutaneous *M. kansasii *infection typically requires skin biopsy for histopathologic examination, AFB staining, and mycobacterial culture. On histopathologic evaluation, cutaneous lesions demonstrate necrotizing granulomatous inflammation and numerous AFB in the absence of peripheral nerve involvement. More recently, next-generation sequencing has gained prominence because of its ability to detect low-abundance and rare pathogens across various sampling sites with high sensitivity [11]. The Genotype Mycobacterium CM assay is a rapid test based on DNA STRIP technology that permits identification of the *M. tuberculosis *complex and commonly encountered NTM to the species level. The sensitivity of the test is greater than 97%, and the specificity is greater than 93.5% [12].

Management of cutaneous *M. kansasii *infection generally follows treatment recommendations established for pulmonary and disseminated forms. *M. kansasii *is intrinsically resistant to pyrazinamide because it lacks a functional pyrazinamidase enzyme needed to convert pyrazinamide to its active form, pyrazinoic acid. Treatment often begins with empiric therapy for *M. tuberculosis *following identification of AFB on staining. The 2020 American Thoracic Society/Infectious Diseases Society of America guidelines recommend initial susceptibility testing for rifampicin to guide treatment decisions. Untreated lesions tend to have a slowly progressive course; however, several reports have demonstrated significant clinical improvement and complete resolution following initiation of appropriate species-directed antimicrobial therapy [13].

For drug-susceptible isolates, the preferred daily regimen is a weight-based, three-drug regimen containing a macrolide with rifampicin and ethambutol. The macrolide component may also be replaced with isoniazid. An intermittently dosed regimen may also be considered. For rifampicin-resistant isolates, a regimen including either a macrolide (azithromycin or clarithromycin) or moxifloxacin plus ethambutol and isoniazid is recommended. Treatment should be continued for at least 12 months [14].

We acknowledge that detailed microbiological culture information regarding this case, including the culture medium, colony morphology, and incubation characteristics, was unavailable because the culture was performed at a peripheral laboratory, and these records could not be retrieved.

## Conclusions

This case emphasizes the importance of considering NTM in HIV-positive patients with atypical cutaneous lesions, particularly in high HIV-prevalence, resource-limited settings where AFB are often presumed to represent *M. tuberculosis*. Empiric anti-TB therapy in patients receiving antiretroviral therapy may increase pill burden and the risk of adverse effects without providing clinical benefit. Accurate species identification and close follow-up are essential to guide appropriate management, ideally through a multidisciplinary approach involving infectious disease and microbiology expertise.
